# Non-Invasive Measurement of Thyroid Hormones in Domestic Rabbits

**DOI:** 10.3390/ani11051194

**Published:** 2021-04-21

**Authors:** Maria Chmurska-Gąsowska, Natalia Sowińska, Sylwia Pałka, Michał Kmiecik, Joanna Lenarczyk-Knapik, Łukasz Migdał

**Affiliations:** 1 Institute of Veterinary Sciences, University Center of Veterinary Medicine JU-UA, University of Agriculture in Krakow, 30-059 Krakow, Poland; maria.chmurska-gasowska@urk.edu.pl; 2 Department of Genetics and Animal Breeding, Faculty of Veterinary Medicine and Animal Science, Poznan University of Life Sciences PULS, 60-637 Poznan, Poland; 3 Department of Genetics, Animal Breeding and Ethology, Faculty of Animal Sciences, University of Agriculture in Krakow, 30-059 Krakow, Poland; sylwia.palka@urk.edu.pl (S.P.); michal.kmiecik@urk.edu.pl (M.K.); lukasz.migdal@urk.edu.pl (Ł.M.); 4 Veterinary Clinic Multivet, Feliksa Konecznego 6/12u, 31-216 Krakow, Poland; j.lenarczyk90@gmail.com

**Keywords:** triiodothyronine, thyroxine, urine, feces, serum

## Abstract

**Simple Summary:**

Thyroid hormones are essential for metabolism, energy homeostasis and reproduction. Hormones can be measured in various biological source materials: blood, urine, feces, saliva, hair, and others. The most common method for assessing hormone levels, including thyroid hormones, is a blood test, but this method has many limitations, especially in the diagnostic process of non-domestic animals. Non-invasive thyroid hormone measurement methods have been developed in the last decade. The aim of our study was to verify the usefulness of thyroid hormone analysis (total thyroxine, total triiodothyronine, free thyroxine, free triiodothyronine) in urine and feces of the domestic rabbit, comparing them with the serum. Results suggest that free triiodothyronine can be accurately and reliably measured in the feces and urine of the domestic rabbit.

**Abstract:**

Thyroid hormones are essential for metabolism, energy homeostasis and reproduction. Hormones can be measured in various biological source materials: blood, feces, urine, saliva and others. The aim of our study was to verify usefulness of thyroid hormone analysis in the urine and feces of the domestic rabbit (*Oryctolagus cuniculus f. domesticus*), comparing them with the serum analyses. Samples were collected from 27 does in the age of 12–14 weeks. Total thyroxine (tT4), total triiodothyronine (tT3), free thyroxine (fT4) and free triiodothyronine (fT3) were tested using the radioimmunological method in serum, feces and urine. The highest concentration of tT4 was found in feces (104.72 ± 59.52 nmol/mg) and the lowest in urine (3.03 ± 3.11 nmol/mL). The highest tT3 concentration was found in blood serum (3.19 ± 0.64 nmol/L) and the lowest in urine (0.31 ± 0.43 nmol/L). The highest concentration of fT4 was observed in feces (43.71 ± 4.79 pmol/mg) and the lowest in blood serum (14.97 ± 3.42 pmol/L). The statistically highest concentration of fT3 (28.56 ± 20.79 pmol/L) was found in urine, whereas the lowest concentration of this hormone was found in feces (3.27 ± 1.33 pmol/mg). There was a positive and statistically significant correlation between serum and urine fT3 (r = 0.76) and a high positive correlation between serum and feces fT3 concentration (r = 0.62). Correlations between concentrations of other thyroid hormones between serum, urine and feces were found to be insignificant. The results suggest that fT3 can be accurately and reliably measured in the feces and urine of the domestic rabbit.

## 1. Introduction

Hormones can be measured in various biological source materials, including blood, saliva, urine, faeces, hair, cerebrospinal fluid, and others. The choice of which is the most appropriate material to sample depends on many complex factors, including the available analytical methods, the type of information sought, the differences in species with respect to the metabolism of steroid hormones and their excretion route, and the availability of the source material [1]. The most common, and by far the most developed, method for assessing hormone levels is to measure it in blood serum [2]. However, this method has many limitations of an organisational nature (taking blood from wild animals can be very dangerous or even impossible), an ethical nature (for most species of animal, subduing the animal in order to take blood is stressful) or a technical nature (in many studies, e.g., concerning reproductive hormones, targets may be secreted over several hours, meaning an exact level cannot be determined at the time of initial secretion; in the case of small laboratory animals, the volume of blood that can be taken is also an important limiting factor for testing) [1,2]. It is important to note that stress caused by subduing and taking blood often distorts the hormone level evaluation. This is particularly evident in the case of adrenocortical hormones (cortisol and corticosterone), the secretion of which significantly increases under the influence of most stressors [3]. 

Since the 1980s, non-invasive hormone measurement techniques have been developed intensively to assess the concentration of hormones and their metabolites in urine, faeces and saliva. These biological materials can be obtained from animals without the need for them to be subdued, and even without any human contact. This is particularly useful in the case of non-domesticated animals, but studies show the benefits of using these methods with domesticated or laboratory animals as well [3]. It would seem that limiting or minimising the contact between animals and humans in order to obtain the test material is generally beneficial in most situations. In the case of non-domesticated animals, it allows for the frequent and regular collection of material, without the need to subdue the animal or put it to sleep. In the case of laboratory animals, it is possible to systematically or almost continuously measure hormones in excretions by using special cages or substrates (e.g., LabSand, Coastline Global Inc., Palo Alto, CA, USA) for their collection. It is also important to note that for some hormones whose secretion is highly pulsatile (e.g., testosterone), the analysis of the metabolic products of these hormones in urine or feces not only gives a more integrated picture, but it is also sometimes necessary when information on the long-term hormonal activity of the male testicles is needed. 

Unfortunately, non-invasive hormone measurement methods also have their limitations. Above all, the metabolism of hormones and their excretion routes are species-specific, which makes it impossible to transfer methods developed on one species to another, even closely related species. In addition, it is also very important to know the detailed physiology of a given species, e.g., knowledge of the length of passage of contents through the digestive tract, in order to properly evaluate and interpret data on the level of hormones and their metabolites in feces. For many species of non-domesticated animals, this knowledge is not available, which can pose some difficulties in interpreting results. It is also problematic to evaluate the hormone metabolites in feces and not the hormones themselves: The excreted metabolites are sensitive to bacterial and environmental degradation, which can quite quickly change their concentrations. It is therefore important that the assessment of hormones in feces be carried out as soon as possible after excretion, which is not always possible in the case of field tests on free-living animals [4]. Attempts are currently underway to develop methods to extract hormones from feces under field conditions. 

Until now, most analyses of thyroid hormones have been based on blood tests. As mentioned above, non-invasive methods have a significant advantage over traditional methods, not only for non-domesticated animals. In the case of thyroid hormones, it should also be noted that, although not directly affecting their concentration, the stress caused during restraint and blood-collection can have a significant impact on animal behaviour (e.g., food intake) and physiology, and thus mask any significant changes in thyroid hormone concentrations [2]. The possibility of measuring the concentration of thyroid hormones in samples of urine and feces has been confirmed in numerous studies which have evaluated the metabolic pathways of these hormones. In one study, radiolabelled isotopic hormones T3 and T4 were administered to rats, and then detected in both urine and feces, confirming both these excretion routes of hormones [5]. 

Thyroid hormones are essential for the growth and development of the body and, being responsible for metabolism, are responsible for maintaining energy homeostasis [6,7]. Triiodothyronine free (fT3) and total (tT3), and the thyroxine fraction free (fT4) and total (tT4), are important for the regulation of heart rate, blood pressure, digestion, muscle work, or thermoregulation [8,9,10]. These hormones play an important role in the development and functioning of the reproductive system [11] and influence the activity of the mammalian immune system [10]. The secretion and metabolism of these endocrine active compounds is subject to very complex regulation of the hypothalamic–pituitary–thyroid axis.

Before choosing a method to measure hormone concentration, it is crucial to check its reliability for each species and biological source material type [12]. The aim of this study was to try to establish a correlation between the concentration of thyroid hormones in urine, feces, and blood serum in the domestic rabbit.

## 2. Material and Methods

### 2.1. Animals

This study focuses on the European rabbit (*Oryctolagus cuniculus f. domesticus*, Popielno White breed). Animals were bred at the Experimental Station of the Department of Genetics, Animal Breeding and Ethology, University of Agriculture in Krakow (registered in the National Animal Breeding Centre under number: K017). Rabbits were kept on bedding in wooden cages (80 cm × 70 cm × 65 cm) of dimensions in accordance with applicable standards, standing in an insulated hall (12 m × 25 m) equipped with a water system (nipple drinkers) and forced ventilation. Animals were kept in a 14 L:10 D system (14 h light and 10 h darkness) with a light intensity of 60 lux. Average temperature on the farm varied from 10 °C to 15 °C, depending on the season. Humidity ranged between 50–60%. Animals had permanent access to water and complete granulated feed, and were under constant veterinary supervision. Cages and feeders were regularly cleaned and disinfected according to the animals’ needs. Rabbits underwent prophylactic treatments including vaccination against rabbit haemorrhagic disease and myxomatosis.

### 2.2. Material

Experiment was conducted under a permit from the Local Ethics Commission (agreement no 267/2018). All samples were taken from 27 females aged 12–14 weeks who were slaughtered. 5 mL of blood for serum samples was collected post-mortem from the external jugular vein (*v. jugularis externa*) by vein cutting and bleeding into PMMA tubes with a coagulation accelerator (Item No.: 22672, FL Medical, Torreglia, Italy). Whole blood was centrifuged for 10 min at Relative Centrifugal Force (RCF) = 2660 (Centrifuge 5430R, Eppendorf, Hamburg, Germany) and pipetted into Eppendorf tubes. Urine was also taken post-mortem by cystocentesis. The amount of urine taken was dependent on how full the bladder was, but not less than 1 mL. Urine was stored in 15 mL Falcon tubes. Feces were taken directly from the last section of the large intestine post-mortem and placed into 30 mL Falcon tubes. All material was immediately frozen at −80 °C.

### 2.3. Preparation and Labelling of Collected Material

Frozen feces were prepared for thyroid hormone extraction according to the method described by Wasser et al. [13] Briefly, 0.1 g thawed feces was mixed with 15 mL of 70% ethanol for 30 min with the aid of a shaker at 1 pulse/s (Compact KS 150, VWR International, part of Avantor, Radnor, PA, USA). Material was then centrifuged for 20 min at 2200 rpm (Sigma 5430 R, Sigma Laborzentrifugen GmbH, Osterode am Harz, Germany). The supernatant was used to determine the concentration of thyroid hormones in feces using a radioimmunological analysis (RIA). 

Thawed urine was shaken and incubated for 30 min at 1 pulse/s (Compact KS 150, VWR International, part of Avantor, Radnor, PA, USA) according to the method described by Yoshida et al. [14] Hormone concentration was measured directly from urine using RIA. 

Serum was prepared and analysed in the same manner as urine, above. 

FT3, tT3, fT4, and tT4 were tested using the radioimmunological method with DIASource kits (catalog numbers of the kits used: fT3-KIPB1579, tT3-KIP1631, fT4-KIPB1363, tT4-KIP1641; DIASource ImmunoAssays S.A., Ottignies-Louvain-la-Neuve, Belgium) on a gamma radiation meter (1740 Wizard, LKB Instruments, Mount Waverley, Victoria, Australia). The test was carried out in accordance with the manufacturer’s instructions attached to the sets in the Isotope Laboratory of the Department of Animal Physiology and Endocrinology of the University of Agriculture in Krakow. 

### 2.4. Statistics

Statistical analysis was carried out using the SAS statistical package (2014), taking into account the consistent effect of the type of biological material used in the model. Significant differences between the averages were determined by Tukey’s test, at a significance level of *p* ≤ 0.05. Pearson correlation coefficients were determined with PROC CORR. 

The following linear model was used:

*Y_ijk_ = µ + FB_i_ + ε_ijk_*

where: *Y_ijk_—*analysed traits (hormone level), *μ—*overall mean, *FB_i_*—effect of *i*-th biological source material (*i* = 1, 2, 3), *ε_ijk_*—residual effect.

SAS (2014). SAS/STAT 13.2 User’s Guide. SAS Institute Inc., Cary, NC, USA.

## 3. Results

Significant differences were found in the concentration of all hormones tested depending on the biological source material. The highest concentration of tT4 was found in feces (104.72 ± 59.52 nmol/mg) and the lowest in urine (3.03 ± 3.11 nmol/mL). Statistically, the highest tT3 concentration was found in serum (3.19 ± 0.64 nmol/L) and the lowest in urine (0.31 ± 0.43 nmol/L). The highest concentration of fT4 was observed in feces (43.71 ± 4.79 pmol/mg) and the lowest in blood serum (14.97 ± 3.42 pmol/L). The statistically highest concentration of fT3 (28.56 ± 20.79 pmol/L) was found in urine, whereas the lowest concentration of this hormone was found in feces (3.27 ± 1.33 pmol/mg) (Table 1).

There was a positive, statistically significant correlation between serum and urine fT3 concentration (0.76) and a high positive correlation between serum and feces fT3 concentration (0.62) (Table 2). Correlations between concentrations of other thyroid hormones between serum and either urine or feces were found to be insignificant (Table 3, Table 4 and Table 5).

## 4. Discussion

The results indicate a high, positive correlation between fT3 concentrations in all of the studied materials, i.e., serum, urine, and feces. fT3 is a hormone with a very low concentration in serum, which does not exceed 1% of tT3 concentration, and is biologically active (i.e., labile). These conditions mean that fT3 represents a diagnostic challenge, yet it most accurately reflects the activity of thyroid hormones in the body. The result reported here is therefore promising for research on methods of evaluating hormonal status in rabbits using non-invasive methods.

The possibility of measuring the concentration of thyroid hormones in samples of urine and feces has been confirmed in numerous studies in different species in which the metabolic pathways of these hormones had been studied. Expression of type I deiodinase, one of three deiodinases responsible for the metabolism of thyroid hormones, is highest in the liver and kidneys. It produces reverse triiodothyronine (rT3) and conjugated forms of thyronine with glucuronates or sulphates that freely mix with bile and are removed with feces [15,16,17]. Metabolites of thyroid hormones in urine are present both in conjugated form, with glucuronic acid, and in unconjugated form [5,17]. Unconjugated forms are free thyroid hormone fractions: fT3 is excreted in the renal tubule; and fT4 is filtered in the glomerulus and partially recovered in the renal tubule [5]. It is known that tT4 is more common in conjugated form with glucuronic acid, while tT3 can be either conjugated with glucuronic acid or with sulphates [18].

It is difficult to draw comparisons to literature on the precise topic addressed in the current study; there is no work of a directly similar nature performed on rabbits. However, other works have assessed metabolism and excretion of hormones somewhat more broadly.

Brown-Grant and Gibson [19] investigated the metabolism of thyroid hormones in rabbits, both endogenous and exogenous, using radiolabelled iodine ^131^I. It was found that the main chemical form of thyroid hormone metabolism is iodide. Following administration of labelled iodide, radioactivity was very low during the first 24 h. During this period, up to 70% of the administered dose appeared in urine. Fecal excretion initially lagged behind urinary excretion, but the curves that were later obtained were similar, with iodine being excreted mainly in organic form (this being true for both endogenous and exogenous hormones). Unfortunately, no correlation has been established between serum hormone concentrations and excretions. Several years later, the Brown-Grant and Tata team performed a similar study on the distribution and metabolism of tT4 and tT3 in rabbits (1961). Radiolabelled hormones were administered intravenously with radioactive iodine ^131^I at a physiological dose, as well as at a dose defined as large. The distribution of hormones was evaluated to assess the validity of the approach of measuring radiation in individual organs. It was found that 30 min after the administration of hormones, the concentration of tT3 tissues was higher than tT4, with tT4 reaching the highest concentration in muscles and the liver. The serum half-life for tT4 was determined as 27 h and for tT3 as 26. For the very large dose of the hormone, the half-life was extended for tT4 to 34 h, and for tT3 to 29 h. The main route of hormone excretion at high doses was found to be via the kidneys.

Similar studies have been carried out on other animal species. In one rat study, isotopically labelled tT3 and tT4 hormones were administered to the animals, and subsequently radiolabelled in both urine and feces, confirming both routes of excretion for these hormones [5]. The duration of hormone removal from blood circulation and excretion in urine or feces was estimated in another rat study. The first radiolabelled tT4 in feces and urine was found after six hours and the concentrations increased for the next five days [20]. In another species—the Steller sea lion (*Eumetopias jubatus*)—following intramuscular injection of thyroid stimulating hormone (TSH), tT3 concentration in feces reached the highest concentration in three of four animals after 48 h [21]. Despite the varying times of occurrence of hormone concentrations in excretions, these studies confirm the possibility of quantitative evaluation of these hormones in urine and feces. Studies carried out by the Wasser team in 2010 show that tT4 is removed with feces in correlation with the circulating storage pool (inactive), while tT3 is directly proportional to the active form of the hormone in serum and its metabolism (Wasser et al., 2010), which makes it more useful in hormone monitoring. 

The work of Wasser et al. [13] and Gesquiere et al. [22] showed yet another aspect that should be taken into account in the methodology of non-invasive testing of thyroid hormones. In herbivores and baboons, the concentration of tT4 in feces was undetectable. Results from the current study confirm this, where it is the active form of the hormone, tT3 in serum, that shows a high correlation with the hormone concentrations in excretions—not tT4. The correlation between serum and fecal concentrations of thyroid hormones is undoubtedly influenced by the rate of passage of food content [23]. It has also been shown that the measurement of thyroid hormone concentrations from feces is subject to a lower error, resulting, for example, from daily fluctuations in mammalian secretion of these hormones. 

A human study by Shakespear and Burke [5] found a positive correlation for total and free thyroxine concentrations between serum and urine. Another human study also showed a high positive correlation between free thyroxine in urine and total thyroxine and free thyroxine in serum [24].

In the 1990s, it was observed that tT3 and tT4, which can already be found in feces in the intestinal area after expulsion with bile, can be recovered by the organism or decomposed by the local microbiota [25]. This is explained by the presence of deiodinase in the intestinal wall, and the ability of bacteria living in the intestinal lumen to inactivate deiodinase [25]. It is also known that some bacteria in the intestine have the ability to cleave the bonds of iodothyronine with glucuronic acid and sulphates [26,27]. As a result of this activity, anaerobic bacteria can recover iodothyronine when there is a deficiency in the body, and thus represent an important component in the metabolism of thyroid hormones.

Scientific studies show that differences in the rate and proportion of excretion of thyroid hormones occur not only between species, but also between individuals, indicating the need for physiological or biological validation of the test. Given that physiological validation is an invasive method, biological validation may provide an acceptable alternative. The time taken for radiolabelled thyroid hormones to appear in feces in the two dogs investigated in the study by Wasser et al. [13] varied significantly. This was explained by the different temperament and activeness of the animals involved. In other studies, an increase in body weight increased the excretion of triiodothyronine in the feces of the Hawaiian monk seal [28], and a decrease in daily rations resulted in a decrease in fecal triiodothyronine concentration in howler monkeys [29].

In addition to the considerations mentioned above, the methodology of collection and extraction of thyroid hormones from urine and feces is important. In the current study, we extracted hormones from feces according to the method used by Wasser et al. [13], who compared the use of ethanol at different concentrations (50%, 60%, 70%, 80%, 90%) and showed that the most appropriate ethanol concentration for thyroid hormones is 70%. Feces should be frozen as soon as possible after collection, due to the proven effects that fecal bacteria have on hormone metabolism [30,31] and the possibility of cross-reacting metabolites appearing [32], which may lead to false results relating to hormones or their metabolites. 

We measured hormone concentrations according to the method of Yoshida [14], i.e., directly from urine without extraction, assuming that there would be no matrix effect in which other substances in the sample might affect the analysis of a given parameter. In their review work, Behringer et al. [2] present the high effectiveness of measuring triiodothyronine from untreated urine. The authors do not rule out the possibility that it might have been wrong to assume a lack of matrix effect in relation to all thyroid hormones. Literature data indicate very good results for thyroid hormone extraction from urine using ethyl acetate [33]. Unfortunately, this method overestimates the concentration of triiodothyronine in relation to thyroxine deionation in urine, as shown by Rogowski and Sirsbaek-Nelsen [34], who used liquid chromatography (Sephadex columns) to separate bound and free thyroxine fractions. Methods of enzymatic hydrolysis are also known [35]. In a study of hormone concentration in urine, it was also recommended to determine the specific gravity of urine and creatinine concentration [36]; this is defined by the relationship between the concentration of the substance dissolved in urine in relation to the amount of water taken up and the volume of urine excreted.

Thyroid hormone levels and their metabolism are known to be influenced by animal age, gender and many other physiological aspects. The results of our studies obtained in a very homologous group (young females) may not be translated in other groups within the tested species, e.g., males or pregnant females. This requires further research.

Over the last twenty years, research into non-invasive methods has accelerated due to its recognition as a possible method for testing free-living animals, whose physiology has so far been poorly understood [2,13,21,22,28,37,38,39]. It is not insignificant, in these ecologically oriented times marked by environmental change, that this type of research is becoming an important element in assessing the human impact on the environment. The assumption made in this study requires further work, and may in future form the basis for a similar study for wild Leporidae. 

## Figures and Tables

**Table 1:** Average hormone concentrations in individual samples.

Hormone	Serum (Mean ± SD)	Urine (Mean ± SD)	Faeces (Mean ± SD)
tT4	72.54 ± 14.99 ^b^ nmol/L	3.03 ± 3.11 ^c^ nmol/L	104.72 ± 59.52 ^a^ nmol/mg
tT3	3.19 ± 0.64 ^a^ nmol/L	0.31 ± 0.43 ^c^ nmol/L	0.83 ± 0.37 ^b^ nmol/mg
fT4	14.97 ± 3.42 ^c^ pmol/L	27.12 ± 14.29 ^b^ pmol/L	43.71 ± 4.79 ^a^ pmol/mg
fT3	10.78 ± 2.81 ^b^ pmol/L	28.56 ± 20.79 ^a^ pmol/L	3.27 ± 1.33 ^c^ pmol/mg

^a, b, c^—within each row, averages marked with different letters differ considerably in terms of significance (*p* ≤ 0.05).

**Table 2:** Phenotypic correlations of fT3 hormone content in the biological materials tested.

Biological Material	Serum	Urine	Faeces
serum	1	0.76 *	0.62 *
urine		1	0.72 *
feces			1

* correlation coefficients are significant (*p* < 0.05).

**Table 3:** Phenotypic correlations of tT4 hormone content in the biological materials tested.

Biological Material	Serum	Urine	Feces
serum	1	−0.06	−0.02
urine		1	−0.51 *
feces			1

* correlation coefficients are significant (*p* < 0.05).

**Table 4:** Phenotypic correlations of tT3 hormone content in the biological materials tested.

Biological Material	Serum	Urine	Feces
serum	1	−0.21	−0.23
urine		1	0.16
feces			1

No significant correlations were found.

**Table 5:** Phenotypic correlations of fT4 hormone content in the biological materials tested.

Biological Material	Serum	Urine	Feces
serum	1	0.03	−0.15
urine		1	0.10
feces			1

No significant correlations were found.

## Data Availability

The data presented in this study are available in this paper.
